# *Angelica gigas* extract inhibits acetylation of eNOS via IRE1α sulfonation/RIDD-SIRT1-mediated posttranslational modification in vascular dysfunction

**DOI:** 10.18632/aging.205343

**Published:** 2023-12-13

**Authors:** Geum-Hwa Lee, Hwa-Young Lee, Young-Je Lim, Ji-Hyun Kim, Su-Jin Jung, Eun-Soo Jung, Soo-Wan Chae, Juwon Lee, Junghyun Lim, Mohammad Mamun Ur Rashid, Kyung Hyun Min, Han-Jung Chae

**Affiliations:** 1 Research Institute of Clinical Medicine of Jeonbuk National University-Biomedical Research Institute of Jeonbuk National University Hospital, Jeonju, Republic of Korea; 2 Non-Clinical Evaluation Center, Biomedical Research Institute, Jeonbuk National University Hospital, Jeonju, Republic of Korea; 3 School of Pharmacy and Institute of New Drug Development, Jeonbuk National University, Jeonju, Republic of Korea; 4 Clinical Trial Center for Functional Foods (CTCF2), Jeonbuk National University Hospital, Jeonju, Republic of Korea; 5 Department of Pharmacology and Institute of New Drug Development, Jeonbuk National University Medical School, Jeonju, Republic of Korea

**Keywords:** *Angelica gigas*, decursin, IRE1α sulfonation, RIDD, SIRT1, vascular dysfunction

## Abstract

*Angelica gigas* NAKAI (AG) is a popular traditional medicinal herb widely used to treat dyslipidemia owing to its antioxidant activity. Vascular disease is intimately linked to obesity-induced metabolic syndrome, and AG extract (AGE) shows beneficial effects on obesity-associated vascular dysfunction. However, the effectiveness of AGE against obesity and its underlying mechanisms have not yet been extensively investigated. In this study, 40 high fat diet (HFD) rats were supplemented with 100–300 mg/kg/day of AGE to determine its efficacy in regulating vascular dysfunction. The vascular relaxation responses to acetylcholine were impaired in HFD rats, while the administration of AGE restored the diminished relaxation pattern. Endothelial dysfunction, including increased plaque area, accumulated reactive oxygen species, and decreased nitric oxide (NO) and endothelial nitric oxide synthase (eNOS) Ser1177 phosphorylation, were observed in HFD rats, whereas AGE reversed endothelial dysfunction and its associated biochemical signaling. Furthermore, AGE regulated endoplasmic reticulum (ER) stress and IRE1α sulfonation and its subsequent *sirt1* RNA decay through controlling regulated IRE1α-dependent decay (RIDD) signaling, ultimately promoting NO bioavailability via the SIRT1-eNOS axis in aorta and endothelial cells. Independently, AGE enhanced AMPK phosphorylation, additionally stimulating SIRT1 and eNOS deacetylation and its associated NO bioavailability. Decursin, a prominent constituent of AGE, exhibited a similar effect in alleviating endothelial dysfunctions. These data suggest that AGE regulates dyslipidemia-associated vascular dysfunction by controlling ROS-associated ER stress responses, especially IRE1α-RIDD/*sirt1* decay and the AMPK-SIRT1 axis.

## INTRODUCTION

Metabolic syndrome (MS) is a group of metabolic disorders linked to multiple risk factors, including hypertension, hyperglycemia, dyslipidemia, obesity, insulin resistance, cardiovascular disease (CVD), and increased risk of type II diabetes mellitus (T2DM). Recent prevalence estimates indicate that approximately 25% of adults have MS [1]. Moreover, high-fat diet (HFD)-induced obesity considerably increases the risk of MS, which is strongly associated with microvascular and macrovascular diseases [2]. These vascular diseases influence the morbidity and mortality in obesity-induced MS [3]. However, the specific mechanisms involved in the development of macrovascular lesions have not yet been fully understood. During oxidative stress, an imbalance between excessive free radical formation and low antioxidant defenses causes cellular and tissue damage. Moreover, the increased production of uncoupled endothelial nitric oxide synthase (eNOS) contributes to higher endothelial oxidative stress, and NADPH oxidase from mitochondrial respiration causes endothelial dysfunction during hyperlipidemia [4]. eNOS can be acetylated, nitrosylated, or phosphorylated to regulate its nitric oxide (NO) synthesis potential, depending on changes in its location or environment [5, 6]. The blood flow disruption induces the expression of molecules involved in atherosclerosis and elevates reactive oxygen species (ROS) levels in endothelial cells (ECs) [7]. Contrarily, steady flow in the arterial tree inhibits arteriosclerosis and increases eNOS-derived NO bioavailability [4, 8]. Further, SIRT1 plays a critical role in regulating cardiovascular function [9]. Also, it is involved in maintaining endothelial health, promoting vascular homeostasis, modulating inflammatory responses, and enhancing resilience against oxidative stress [10]. The enzyme activates eNOS by deacetylation, thus enhancing NO production via vascular homeostasis. In the rat thoracic aorta, acetylcholine-induced NO production was found to be substantially affected by the overexpression of the adenovirus-induced dominant negative form of the SIRT1 mutant [11]. This study strongly suggests that the expression and acetylation activity of eNOS are regulated by SIRT1. Multiple molecular mechanisms promote hyperlipidemia-induced vascular dysfunction via affecting the protein-folding capabilities of endoplasmic reticulum (ER) chaperones and foldases [12]. Similarly, several molecules such as resveratrol, epigallocatechin gallate, and α-lipoic acid influence and play a critical role in mitigating HFD-induced vascular dysfunction [13–15]. Regardless, innovations in functional food products necessitate a thorough understanding of the mechanisms underlying their beneficial role.

*Angelica gigas* Nakai (AG) is a traditional medicinal herb, is garnering scientific attention for its potential in addressing a variety of health conditions. Recent studies highlight the pharmacological properties of AG, including its antioxidant, hypoglycemic, and lipid-lowering capabilities [16]. Notably, decursin, a key compound in AG, has been found to exhibit a range of biological and functional roles, which underscore its potential applications in therapeutics [17]. Furthermore, AG extracts (AGE) have been demonstrated lipid-modulating properties with fewer side effects compared to conventional lipid-lowering compounds, suggesting a potential role in managing metabolic disorders [18]. Given the link between HFD-induced endothelial dysfunction and hyperlipidemia, there is a growing interest in the ability of AG to restore endothelial health and regulate vascular function. Consequently, the primary aim of this study is to examine the inhibitory effects of AGE on dyslipidemia-associated vascular dysfunction, with a focus on its potential mechanisms of action.

## RESULTS

### AGE improves Ach-induced vascular relaxation

**Figure 1:**
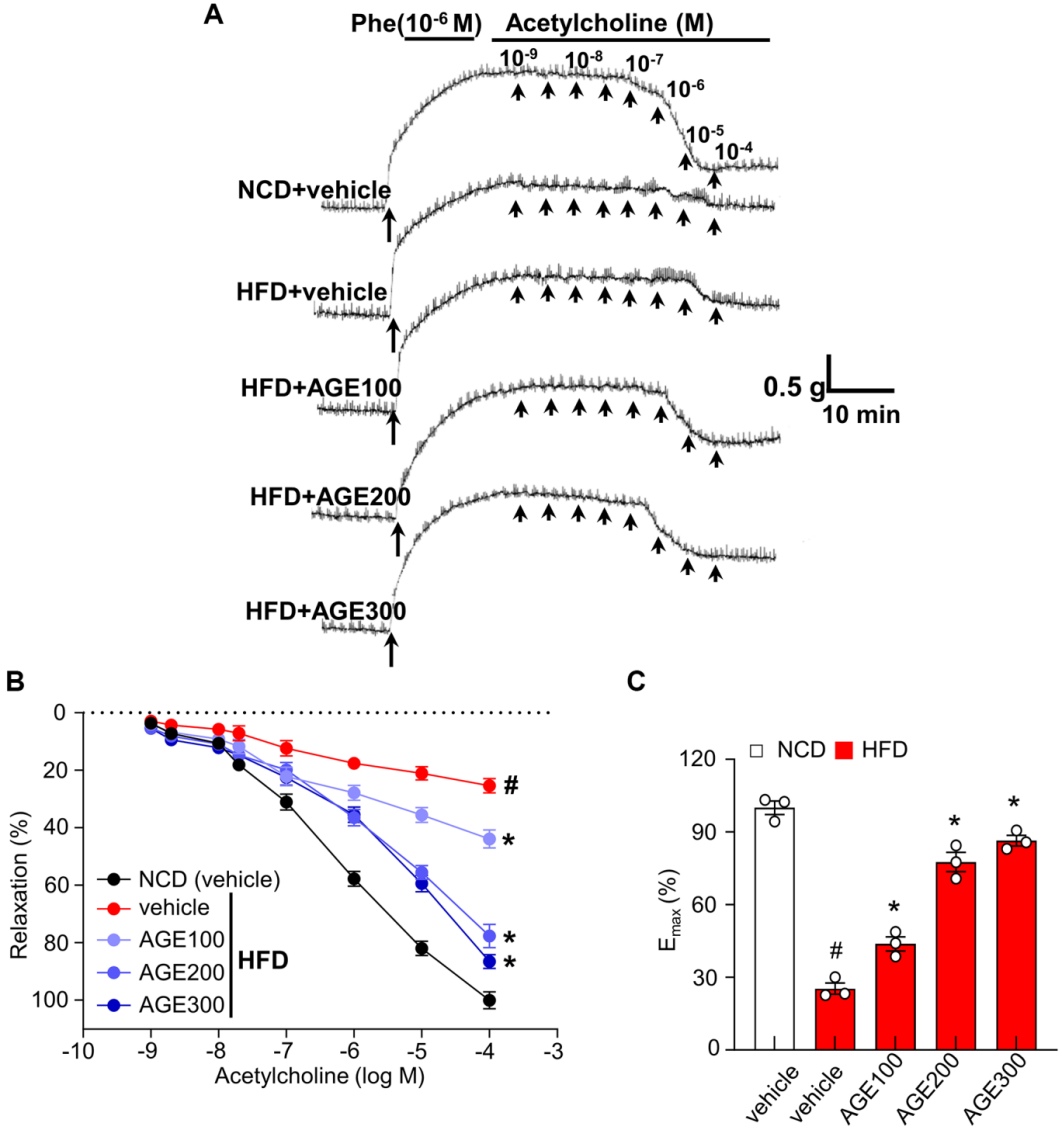
**AGE enhances endothelial relaxation in aorta rings.** (**A**) Representative traces under specified conditions. (**B**) Percentage of relaxation proportional to Ach concentration. (**C**) Maximum relaxation by Ach (10^−4^ M) in aorta from Figure 1B. Results are expressed as percent relaxation ± SEM. (*n* = 4; ^#^*p* < 0.05 vs. control). Abbreviations: Phe: phenylephrine; NCD: normal chow diet; HFD: high fat diet; AGE: *Angelica gigas NAKAI* extract.

In the contractile experiment with Sprague-Dawley rat thoracic aorta rings, the aorta from normal chow diet-feeding (NCD) rats showed dramatic contraction under the 10^−6^ M of phenylephrine (Phe) and subsequently showed gradual relaxation under the 10^−1^ folded concentrations of acetylcholine (Ach) from 10^−9^ M to 10^−4^ M. The aorta from HFD rats showed significantly less contraction and relaxation than that from the NCD rats under the same procedures, whereas the aorta from AGE-administered HFD rats improved the reduced capability of contraction and relaxation in the administered AGE dose-dependent manner (Figure 1A). The vasorelaxation percentage (Figure 1B) and the maximum relaxant effect of Ach (10^−4^ M); a plateau vasorelaxation state (E_max_) (Figure 1C) showed that AGE improved Ach-induced vasorelaxation capability, a key measurable parameter for vascular function *ex vivo* was significantly recovered in the administered AGE dose-dependent manner.

### AGE reduces the size of carotid atherosclerotic plaque and recouples eNOS *in vivo*

**Figure 2:**
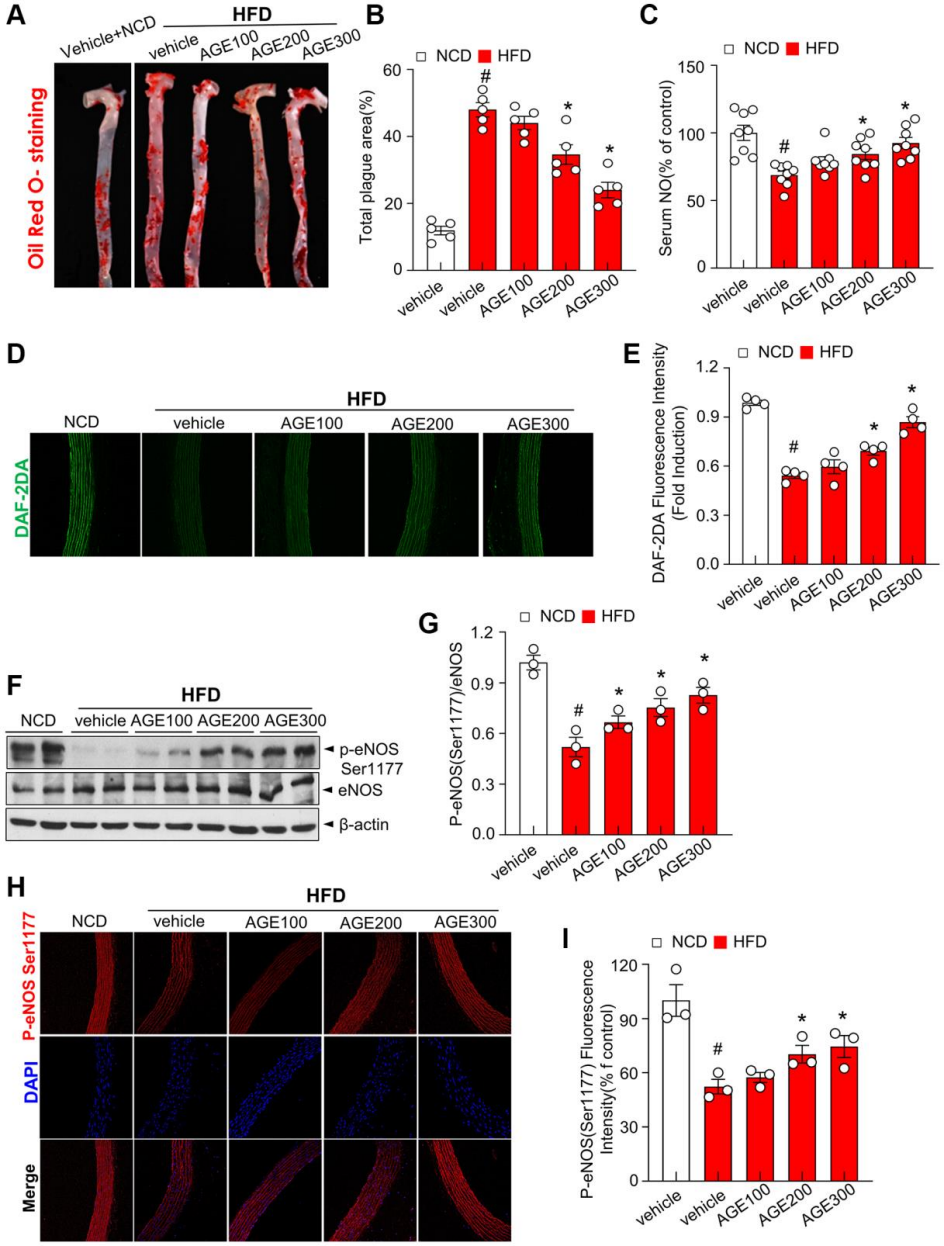
**AGE improves eNOS activity in hyperlipidemic conditions.** (**A**) Representative images of aorta surface stained with Oil Red O and atherosclerotic lesion area in the indicated groups. (**B**) Quantification of atherosclerotic lesion area (plaque area). (**C**) Serum NO levels in the indicated experimental groups. Data shown are relative to serum NO levels in control group. (**D**) Fluorescence microscopic detection of DAF-2DA staining for endothelial NO levels in the aorta and (**E**) its quantification. (**F**) Immunoblotting of eNOS and phosphorylated eNOS in aorta. (**G**) Quantitative analysis of phospho-eNOS based upon the eNOS expression. (**H**) Representative immunofluorescence images showing p-eNOS staining at Ser1177 (p-eNOS) in aortic sections. (**I**) Quantification of phosphorylation of eNOS at Ser1177. Values are presented as mean ± SEM (*n* = 4~9, ^#^*p* < 0.05 vs. the NCD group; ^*^p < 0.05 vs. the HFD group). Abbreviations: NCD: normal chow diet; HFD: high fat diet; AGE: *Angelica gigas NAKAI* extract.

Next, we examined the effect of AGE on oxidative stress and atherosclerosis progression in HFD rats with dyslipidemia. Under the HFD condition, body weights were unaltered in the AGE-administered rats compared with vehicle-administered group (Supplementary Figure 1). Oil Red O-positive staining and total plague area, the representative *ex vivo* markers for oxidative stress and atherosclerosis progression, were significantly increased in the aorta from HFD rats, whereas these parameters were significantly reduced in the aorta by AGE supplementation (Figure 2A, 2B). Serum NO levels were significantly reduced in HFD groups whereas the AGE group showed higher serum NO levels compared to the HFD vehicle group (Figure 2C). The fluorescence intensity of DAF-2DA, a fluorescence dye for NO in the aorta was decreased in the aorta from HFD rats whereas the intensity was significantly recovered AGE supplementation (Figure 2D, 2E). AGE supplementation decreased the phosphorylation of eNOS at Ser-1177 whereas the phosphorylation was significantly recovered in the aorta in the HFD rats (Figure 2F, 2G). Immunofluorescence analysis also confirmed the restoration of eNOS Ser-1177 phosphorylation with AGE treatment (Figure 2H, 2I).

### AGE alleviates endothelial vascular dysfunction and oxidative stress in HFD-induced hyperlipidemic rats

**Figure 3:**
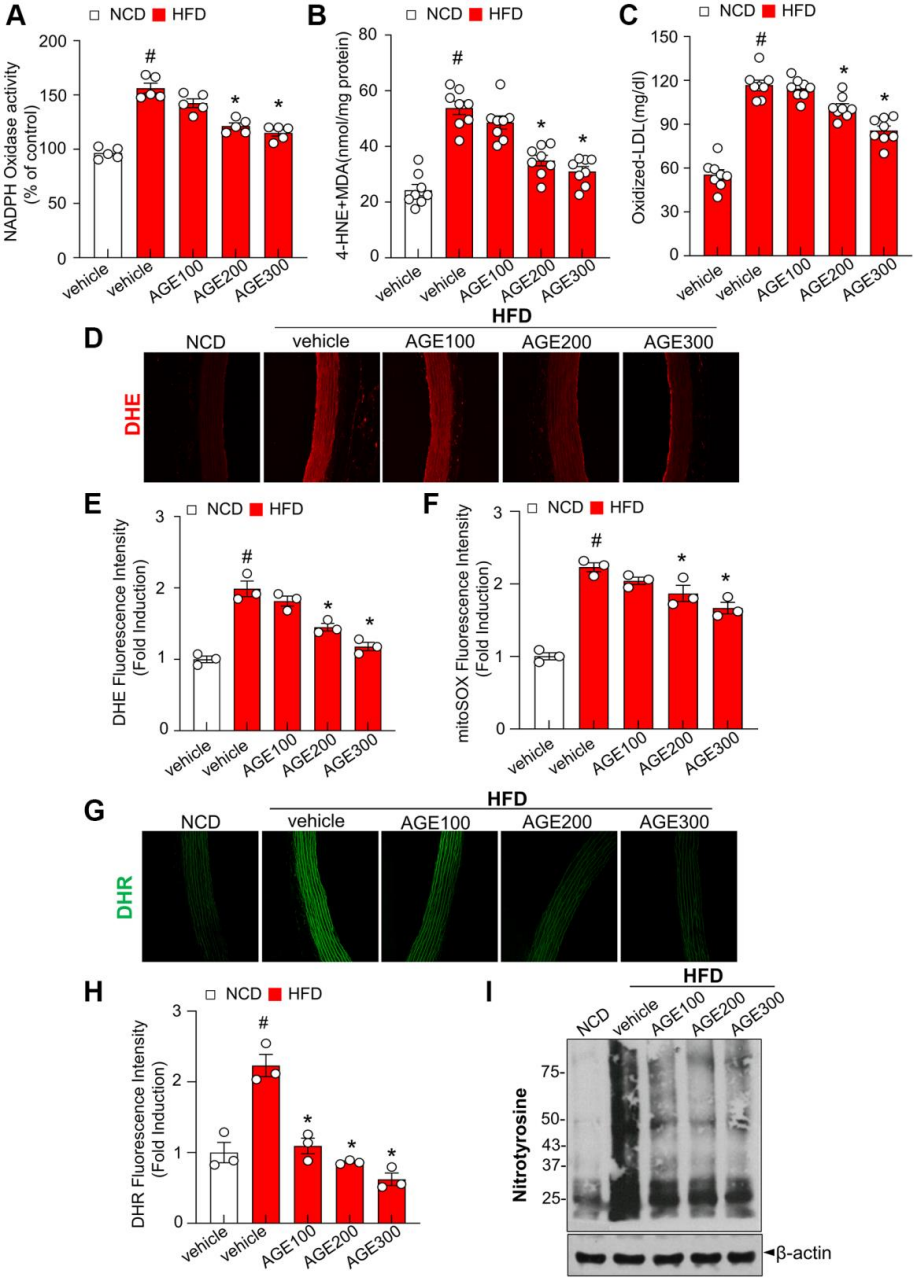
**AGE inhibits oxidative stress in hyperlipidemic rats.** (**A**) NADPH oxidase activity determined via lucigenin chemiluminescence assay. (**B**) 4-HNE + MDA level was measured as described in Materials and Methods. (**C**) Oxidized low-density lipoprotein (Ox-LDL) cholesterol content in plasma. (**D**) Representative images of DHE staining for ROS production and (**E**) quantification of DHE fluorescence intensity for ROS levels in aortas. (**F**) Quantification of mitoSOX fluorescence intensity for mitoROS levels in aortas. (**G**) Representative images of DHR staining for peroxynitrite in aortas and (**H**) quantification of DHR fluorescence intensity for peroxynitrite levels in aortas. (**I**) Nitrotyrosine analysis was performed as was described in Materials and Methods. Values are presented as mean ± SEM (*n* = 4~9, ^#^*p* < 0.05 vs. the NCD group; ^*^p < 0.05 vs. the HFD group). Abbreviations: NCD: normal chow diet; HFD: high-fat diet; AGE: *Angelica gigas NAKAI* extract.

The NADPH oxidase system is a major source of ROS [19]. In the present study, NADPH oxidase activity in the HFD group was significantly higher than that in the NCD group (Figure 3A). However, AGE supplementation reversed this effect in a dose-dependent manner. Similarly, AGE reduced lipid peroxidation in the aorta from HFD rats (Figure 3B). Additionally, the production of oxidized LDL (ox-LDL), an atherosclerosis-linked ROS generator, was examined. As expected, ox-LDL was highly elevated in the HFD group and significantly decreased under the AGE supplementation (Figure 3C). After 8 weeks of treatment with AGE, serum levels of fasting glucose, TG, TC, and LDL were significantly reduced in the AGE-administered HFD group compared to the HFD-vehicle group (Supplementary Figure 2). DHE fluorescence throughout the aortic wall in the HFD group was higher than that in the NCD group whereas AGE supplementation induced a dose-dependent reduction of DHE fluorescence (Figure 3D, 3E). The MitoSox fluorescence signal, an indicator of superoxide anions [20], was increased in the aortic rings from HFD rats, whereas AGE supplementation downregulated the superoxide formation in the aortic rings (Figure 3F). The fluorescent probe DHR reacts directly with peroxynitrite-derived free radicals but not with O_2_^−^ or NO [21]. The fluorescence of DHR was highly increased in the aorta from HFD group compared with NCD group whereas AGE-administered HFD group markedly reduced the levels of DHR compared with the HFD group (Figure 3G, 3H). Consistently, the nitrotyrosine level increased in the HFD condition compared with NCD condition whereas AGE administration under HFD markedly reduced the nitrotyrosine level compared with the HFD condition (Figure 3I). In contrast, aortic wall thickness was not significantly reduced in AGE-administered HFD group compared with HFD group (Supplementary Figure 3).

### AGE maintains SIRT1-eNOS deacetylation signaling pathways through the regulatory effect against the IRE1α-RIDD; *sirt1* decay axis

**Figure 4:**
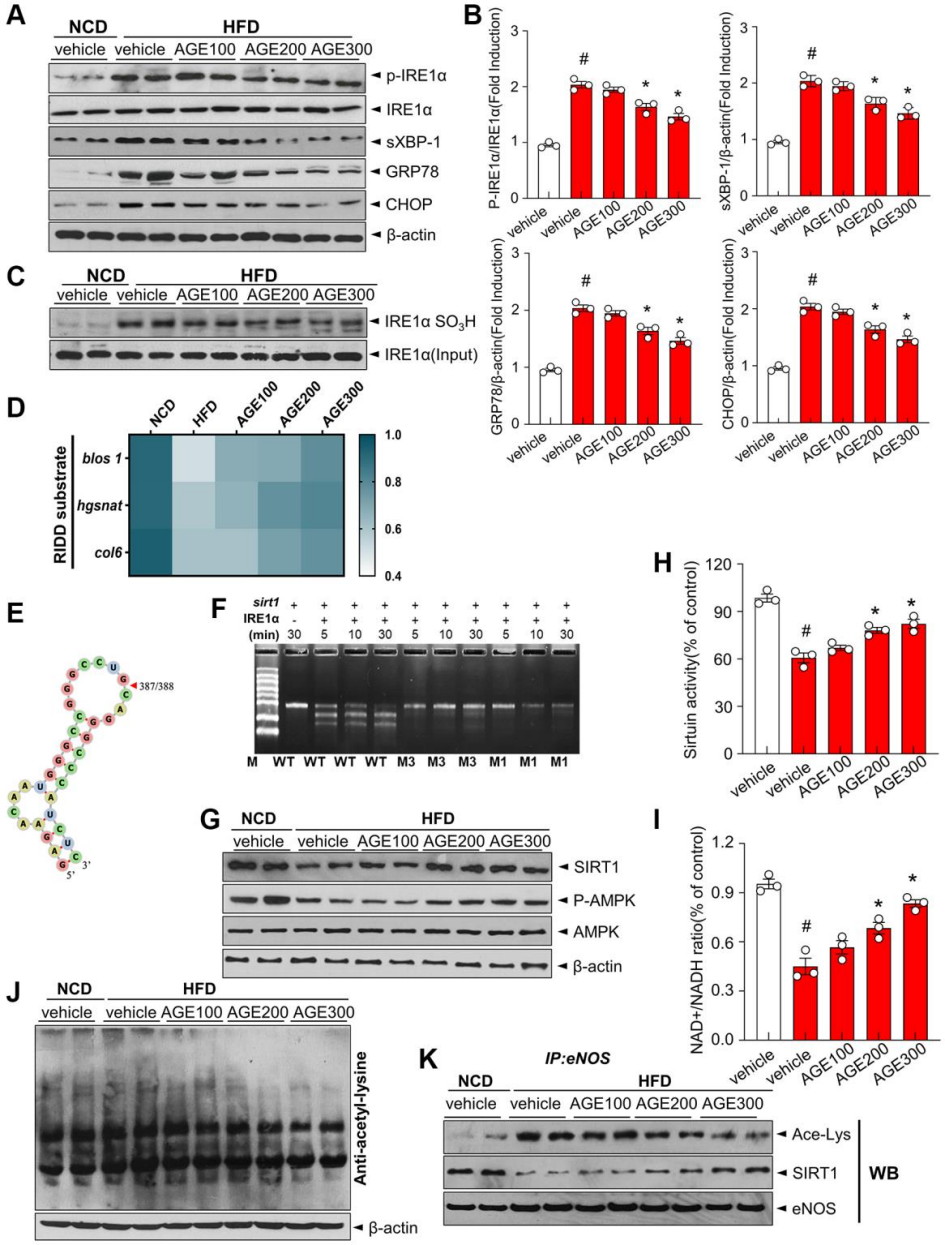
**AGE regulates ER stress and IRE1α sulfonation in vascular dysfunction model.** (**A**) Immunoblotting of p-IRE1α, IRE1α, sXBP-1, GRP78, CHOP and β-actin expressions in aorta and (**B**) respective quantitative analysis of protein expressions. (**C**) Immunoblotting with anti-IRE1α SO_3_H and IRE1α antibodies in aortas. (**D**) Heatmap depicting mRNA expression of genes identified as RIDD substrates in the aorta. (**E**) RNA fold prediction in the secondary structure of mRNA fragments of *sirt1*. (**F**) *In vitro* cleavage assay using 1.5% denaturing agarose gel. Sirt1 mRNA cleaved by IRE1α with its two mutant mRNAs during the indicated times. (**G**) Immunoblotting of SIRT1, p-AMPK, AMPK, and β-actin in aorta from the indicated groups. (**H**) Sirtuin activity and (**I**) NAD^+^ and NADH levels were measured, and NAD^+^/NADH ratios were quantified. (**J**) Acetylated lysine levels and β-actin expression were determined using immunoblotting. (**K**) eNOS was immunoprecipitated from aorta tissues from the indicated groups, and its acetylated-lysine level was analyzed via immunoblotting with anti-acetyl-lysine and anti-SIRT1 antibody. Data are presented as mean ± SEM (*n* = 3, ^#^*p* < 0.05 vs. NCD, ^*^p < 0.05 vs. HFD). Abbreviations: NCD: normal chow diet; HFD: high-fat diet; AGE: *Angelica gigas NAKAI* extract.

The ER stress response is a potential therapeutic target for vascular disorders [22], Hence, ER stress markers, such as p-IRE1α, sXBP-1, GRP78, and CHOP, were evaluated to determine the impact of AGE on ER stress. In the HFD group, ER stress markers were elevated, whereas AGE regulated their expression (Figure 4A, 4B). Furthermore, phosphorylation of IRE1α, subsequent XBP-1 splicing, and expressions of GRP78 and CHOP were inhibited in the AGE-treated HFD group. IRE1α sulfonation was highly increased in the HFD group whereas that was significantly inhibited in the AGE-administered HFD group (Figure 4C). Using real time PCR, we confirmed the regulated IRE1α-dependent decay (RIDD) pattern, showing the degradation of *blos, hgsnat*, and *col6* mRNA, which was increased in the HFD group but was inhibited in the AGE-administered HFD group (Figure 4D). Sequence analysis confirmed that *sirt1* contains the RIDD sequence ‘CUGCAG,’ which can form an RNA double helix (Figure 4E). *Sirt1* was highly decayed in the presence of IRE1α in a time-dependent manner and the decay was inhibited in the mutants of *sirt1*; M1 (CUUCAG) and M3 (CUGUAG), indicating the role of *sirt1* as a substrate of RIDD (Figure 4F). Consistently, SIRT1 protein expression in the HFD group was decreased compared with the NCD group. Further, the expression was recovered in the AGE-administered HFD group compared with the HFD group (Figure 4G). AMPK phosphorylation was also lower in the HFD group than in the NCD group, whereas AGE considerably recovered the phosphorylation. SIRT1 enhances eNOS enzymatic activity via deacetylation [21]. In the endothelium, SIRT1 functionally interacts with eNOS [9]. In the present study, sirtuin activity, and the mean aortic NAD^+^/NADH ratio were lower in the HFD group than in the NCD group (Figure 4H, 4I). However, AGE supplementation restored sirtuin activity and NAD^+^/NADH ratio. Furthermore, acetyl-lysine levels were significantly increased in the HFD group but were regulated by AGE (Figure 4J). Additionally, eNOS acetylation was higher in the HFD group but was controlled by AGE (Figure 4K).

### AGE and decursin, a major component of AGE, attenuate oxidized LDL-induced oxidative stress in HUVECs

**Figure 5:**
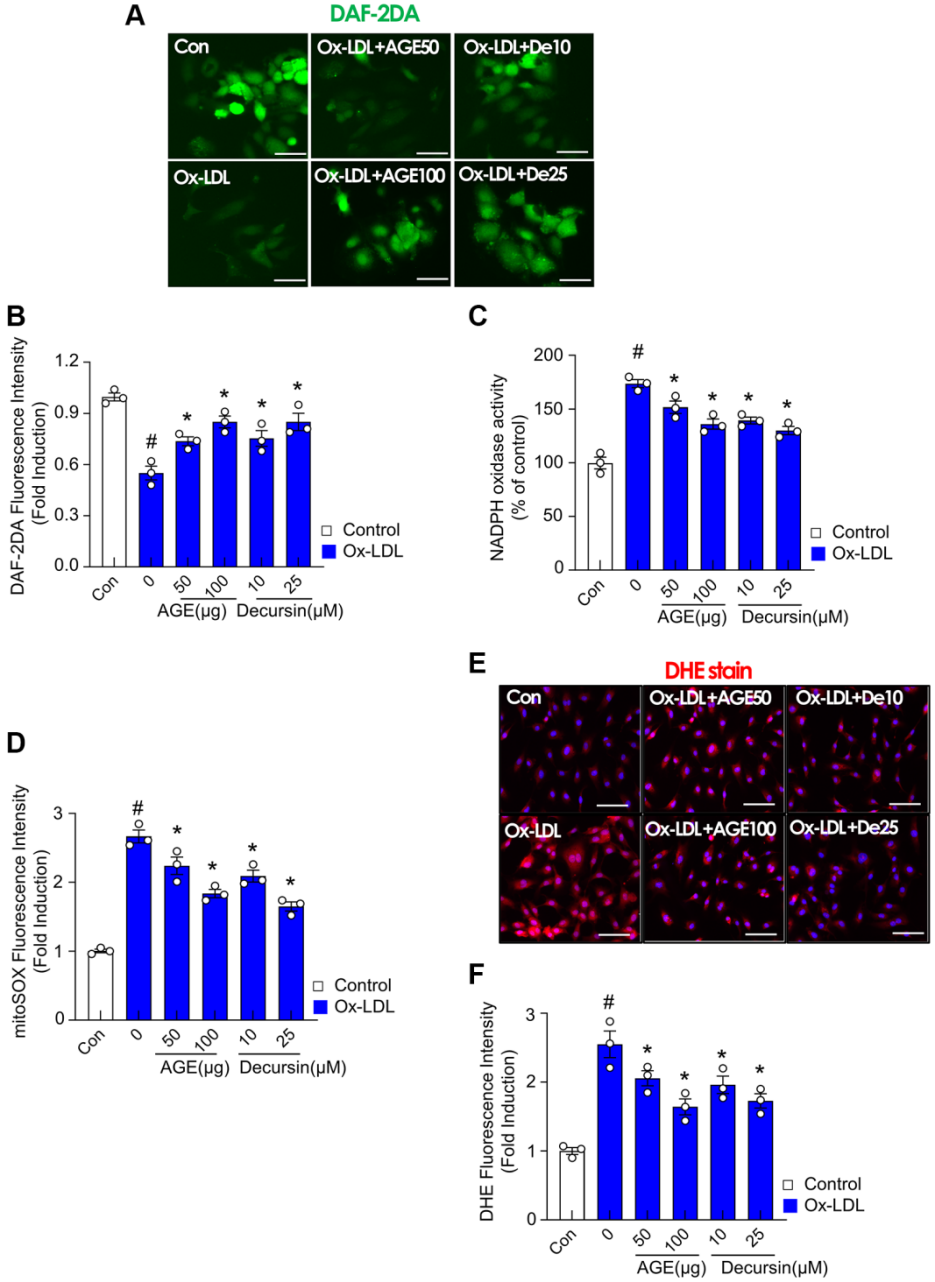
**AGE improves endothelial cell functions in Ox-LDL-treated HUVECs.** HUVECs were treated with decursin, or AGE, and then incubated with Ox-LDL for another 48 h. (**A**) NO production and (**B**) quantification of DAF-2DA fluorescence intensity. (**C**) NADPH oxidase activity was determined using lucigenin chemiluminescence assay. (**D**) Fluorescence intensity for mitoSOX levels was quantified in HUVECs. (**E**) Representative images of DHE staining for ROS production and (**F**) quantification of fluorescence intensity for ROS levels in HUVECs. ^#^*p* < 0.05 compared to the control group; ^*^p < 0.05 compared to the Ox-LDL only treated group. Values are presented as mean ± SEM (*n* = 3). Abbreviations: NO: nitric oxide; DAF-2DA: diaminofluorescein-2 diacetate; Scale bar: 100 μm.

Oxidative stress was examined to understand the effect of AGE on the endothelium. Endothelial dysfunction develops due to abnormalities in NO production by the vascular endothelium [23]. In this study, a DAF-2DA probe was used to detect endothelial NO production. NO reduction by ox-LDL was reversed by AGE and decursin (Figure 5A, 5B). Similarly, AGE and decursin antagonized ox-LDL-induced NADPH oxidase activity (Figure 5C). MitoSOX fluorescence was also highly increased in ox-LDL-treated HUVECs than in the control group (Figure 5D). However, the treatment with AGE and decursin inhibited mitoSOX fluorescence. Additionally, DHE staining confirmed the inhibitory effects of AGE and decursin against ROS formation in HUVECs (Figure 5E, 5F).

### AGE inhibits acetyl-lysine eNOS-associated IRE1α sulfonation/RIDD-*sirt1* in HUVECs

**Figure 6:**
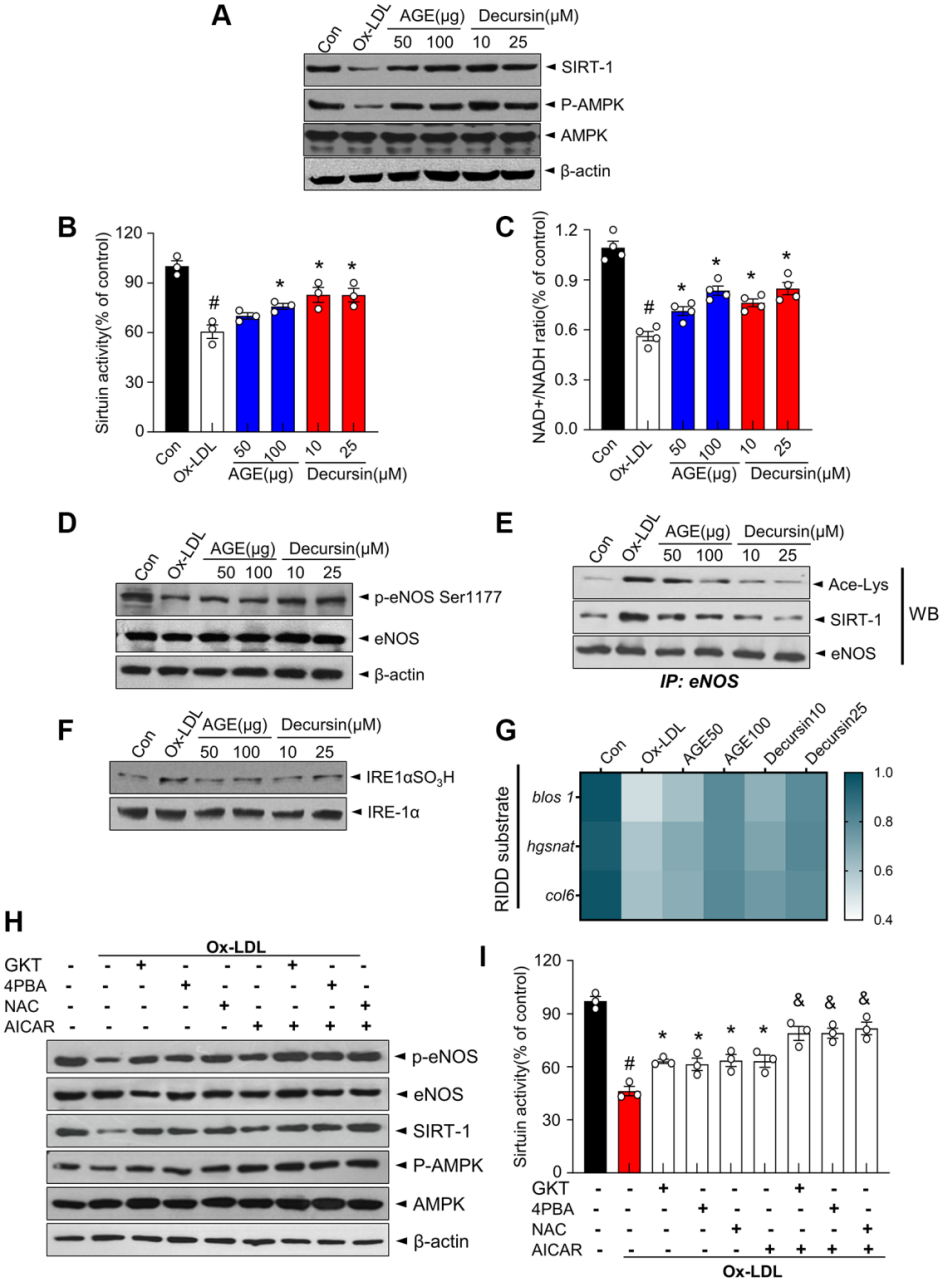
**AGE improves endothelial cell functions in OxLDL-treated HUVECs.** The indicated concentrations of AGE was treated in Ox-LDL-treated HUVECs for 48 h. (**A**) Immunoblotting was performed with anti-SIRT1, p-AMPK, AMPK, and β-actin antibodies in HUVECs. (**B**) Sirtuin activity and (**C**) NAD+ and NADH levels were analyzed, and NAD+/NADH ratios were quantified. (**D**) eNOS phosphorylation at Ser1177 and expression of total eNOS and β-actin in HUVECs were determined using immunoblotting. (**E**) eNOS was immunoprecipitated from HUVEC lysate, and immunoblotting was performed with anti-acetyl-lysine, SIRT1 and eNOS antibodies. (**F**) Immunoblotting was performed with anti-IRE1α SO3H and IRE-1α antibodies. (**G**) Heatmap depicting mRNA expression of the genes identified as RIDD substrates in HUVECs. (**H**) Ten μM GKT, 5 mM 4PBA, 1 mM NAC, or 1 mM AICAR were pre-treated in Ox-LDL-treated cells for 48 h. Immunoblotting was performed with anti-p-eNOS, eNOS, SIRT1, p-AMPK, AMPK, and β-actin antibodies and (**I**) sirtuin activity was measured, as described in Materials and Methods. ^#^*p* < 0.05 compared to the control group; ^*^p < 0.05 compared to the Ox-LDL only treated group. ^&^*p* < 0.05 compared to the each counter AICAR not treated group. Values are presented as mean ± SEM (*n* = 3). Abbreviation: AGE: *Angelica gigas NAKAI* extract.

Under AGE and decursin, the expression and activity of AMPK-SIRT1 and IRE1α-RIDD/*sirt1* decay signaling were examined in ox-LDL-treated HUVECs. First, AMPK phosphorylation and SIRT1 expression decreased in ox-LDL-treated cells whereas they were recovered in AGE- or decursin-treated cells (Figure 6A). Consistently, AGE and decursin restored the decreased sirtuin activity and NAD^+^/NADH ratio in ox-LDL-induced HUVECs (Figure 6B, 6C). Next, eNOS phosphorylation status was examined under AGE- or decursin-treated conditions. Ox-LDL significantly reduced the phosphorylation of Ser-1177, while AGE and decursin recovered the eNOS Ser1177 phosphorylation in HUVECs (Figure 6D). Furthermore, eNOS acetylation and SIRT1-associated with eNOS were significantly increased in ox-LDL-treated cells, whereas AGE or decursin decreased these levels to baseline (Figure 6E). The sulfonation of IRE1α was highly increased and the gene expressions of substrates of RIDD*, blos1, hgsnat* and *col6* were decreased in ox-LDL-treated cells, whereas AGE or decursin restored these levels (Figure 6F, 6G). Next, ox-LDL-induced HUVECs were treated with GKT137831; Nox4 inhibitor, 4-PBA; ER stress inhibitor, N-acetylcysteine; ROS scavenger, and AICAR; AMPK activator to show the involved mechanism of AGE and decursin against endothelial dysfunction. eNOS Ser1177 phosphorylation, SIRT1 expression, and AMPK phosphorylation and sirtuin activity decreased in ox-LDL-treated cells (Figure 6H, 6I). They were partially restored by GKT137831, 4-PBA, and NAC whereas they are more significantly restored by the additional treatment of AICAR compared with the mentioned single agent-treatment, indicating ER stress and AMPK pathways are working independently on SIRT1-eNOS signaling in endothelial system.

### Analysis of compounds in AGE

AGE components were analyzed to determine the influence of AGE on endothelial function. Decursin is a major component of AGE. Thus, we considered decursin as an effective component and used it as a standard component to determine AGE quality. Supplementary Figure 4A shows the structure of decursin. Supplementary Figure 4B, 4C also show chromatogram data for AGE linked to the decursin standard.

## DISCUSSION

The present study demonstrates the beneficial effect of AGE on hyperlipidemia, oxidative stress, NO bioavailability, and associated endothelial contractility in the thoracic aorta of HFD-induced hyperlipidemic rats. The protective effects of AGE are attributed to decursin, a major functional compound in AGE. Decursin controls dyslipidemia-associated vascular dysfunction by regulating ROS-associated ER stress responses, especially IRE1α-RIDD/*sirt1* decay and the AMPK-SIRT1 axis.

In this study, the impact of AGE on contractility was investigated to define its characteristic features of endothelial relaxation. In this specific experiment, we demonstrated that AGE improved the Ach-induced vasorelaxation (Figure 1A–1C). Specifically, atherosclerotic lesions in the aortas of mice fed HFD, which exhibit several characteristic features of atherosclerosis [24, 25], were effectively improved in the presence of AGE. Notably, a dose-dependent response was observed, a crucial phenomenon for elucidating the clinical mechanisms underlying vasorelaxation effects. This study built upon a recently reported method for quantifying atherosclerotic lesions in mouse aortas [26], which employed Sudan IV staining to measure the area of gross atherosclerotic plaques throughout the entire aorta. The research highlighted the role of lipid dysmetabolism-induced ROS and its association with eNOS uncoupling. Endothelial vasorelaxation is based on NO-eNOS phosphorylation, in which ROS, including superoxide anions, induced an uncoupling effect on eNOS activity [27]. Interestingly, our observations revealed the beneficial impact of AGE on eNOS phosphorylation and NADPH oxidase activity, a typical ROS-generating system (Figures 2F–2I, and 3A). The study outcomes suggest that hypermetabolism-associated ROS affects eNOS uncoupling and associated vascular dysfunction in HFD conditions. However, AGE also regulates eNOS coupling and NO release via IRE1α phosphorylation and sulfonation (Figures 2C, 4A–4D). In cell-based physiology, ROS are transferred to subcellular organelles, including the critically important ER, a key organelle that facilitates protein folding and a chaperone-based electron transfer system. Contrary to the adaptive ER stress response, pathological ER stress affects the redox balance, influencing post-translational modifications (PTMs) of proteins and subsequent changes in protein function. Here, IRE1α, an ER stress representative sensor, was highly phosphorylated and sulfonated in the HFD stress state, while AGE regulated the PTMs of the protein and associated XBP-1 splicing and RIDD phenomena (Figure 4A–4F). Generally, the IRE1α-sXBP-1-based chaperone gene transcription axis explains the adaptive ER stress response, whereas sulfonation describes the chronic/severe/pathologic ER stress response [28]. Specifically, endogenous and exogenous ROS play an essential role in oxidizing cysteine residues involved in sulfonation [29]. The IRE1α PTM-linked signaling axis, identified as RIDD, has multiple substrates, including proteins relevant to energy metabolism. *Sirt1* is an IRE1α sulfonation-linked RIDD target that carries a RIDD target sequence [30] (Figure 4E, 4F). Our PCR analysis of *blos 1, Hgsnat,* and *Col6* in OxLDL-treated HUVECs shows mRNA degradation, a finding reflecting RIDD. The RIDD targets are enriched for mRNAs containing a cleavage site with a consensus sequence (CTGCAG) and a predicted secondary structure similar to the conserved Ire1α recognition stem-loop of the Xbp1 mRNA [31, 32], the common characteristic sharing with the mentioned genes including *sirt1.* Recently it has been also reported that sulfonation-regulated RIDD-*sirt1* decay was also highly increased under diabetic conditions [33]. Consistently, the decay of *sirt1* mRNA as a RIDD target was also defined in hyperlipidemic condition here, affecting the disturbed sirtuin activity and NAD^+^/NADH ratio and eNOS activity. The ER stress-associated mechanism may have contributed substantially to the ability of AGE to improve obesity and vascular dysfunction.

AMPK inhibits inflammatory responses by controlling several downstream signaling pathways, including SIRT1 [34]. Interestingly, AGE supplementation reversed the p-AMPK and SIRT1 downregulations in HFD rats (Figure 4G). Additionally, the anti-atherosclerotic effect of AGE may depend on SIRT1, which is highly expressed in human vascular endothelial cells and plays a vital role in regulating endothelial and vascular functions. Upregulation of SIRT1 expression or increase in the activity inhibited endothelial dysfunction stimulated by oxidative stress [35]. Additionally, SIRT1 enhances eNOS enzymatic activity via deacetylation. eNOS is physically associated with SIRT1 in the endothelium. Inhibition of SIRT1 activity enhances eNOS acetylation in the calmodulin-binding domain at lysine residues 496 and 506 [10]. In this study, we focused on the efficacy of AGE in restoring SIRT1 expression and its deacetylation activity. Furthermore, higher eNOS acetylation in hyperlipidemia is associated with an increased risk of cardiovascular disease [35]. AGE reversed this specific effect and improved eNOS-SIRT1 interaction (Figure 4K). These observations indicate a protective effect of the SIRT1-eNOS-NO axis against endothelial dysfunction. Moreover, SIRT1 prevents endothelial dysfunction via deacetylation of PGC-1α and PPARα, which reduces NADPH oxidase activity and improves NO activation [10]. Moreover, high expression of SIRT1 regulates angiogenic function via eNOS-derived NO production, contributing to vascular growth and its function [35]. Thus, eNOS and NO bioavailability disruption define endothelial dysfunction and have been linked to the pathogenesis of hyperlipidemic cardiovascular complications. Even if we observed a positive effect on hyperlipidemia, oxidative stress, NO bioavailability, and vascular function, this experiment has certain limitations, i.e., *ex vivo* vascular relaxation test instead of *in vivo* vascular image etc. This study was based on previous reports that documented the positive effects of AGE against metabolic dysfunction and inflammatory diseases [36, 37]. One of the major components of Angelica gigas (AG), decursin [38], has demonstrated significant consistency with the *in vivo* system in an *in vitro* endothelial cell system, as evident in Figures 5, 6. These figures illustrate its control over ROS and its ability to counteract eNOS uncoupling effects. Furthermore, recent research has established the maintenance of AMPK activity in the presence of decursin and decursinol angelate-rich AGE [16]. What sets this study apart from existing reports on decursin is its focus on the control of ER stress, contributing to the maintenance of the core metabolic switcher SIRT1 and eNOS activity. This study demonstrates the pivotal role of AGE in modulating vascular function, including vasorelaxation and oxidative stress. Additionally, AGE could induce NO synthesis and maintain the ER folding state by regulating ROS production, NADPH oxidase levels, and ER stress responses. Specifically, ER stress responses, such as IRE1α phosphorylation, sulfonation, and its related RIDD/*sirt1* decay, and the relatively known AMPK-SIRT1-eNOS deacetylation axis all play a crucial role in the AGE effect on vascular dysfunction. Ultimately, this study presents clearly evidence that AGE is a promising natural product-based functional food/herbal medicine candidate for preventing or regulating hyperlipidemic cardiovascular complications.

## MATERIALS AND METHODS

### Preparation and analysis of AGE

Fresh AG roots were obtained from Jinbu, Pyeongchang-gun, Gangwon-do, Republic of Korea. The roots of fresh AG were chopped (20 kg), extracted with 95% ethanol (100 L), and incubated for 4 h at 45°C in a DH-M03 accelerated Solvent Extractor (D.M. ENGINEERING Corporation, Siheung, Republic of Korea). The final yield was 4 kg (1/5th of the original root), and the AGE contains 13% decursin. The prepared AGE was analyzed using an HPLC system (Agilent, Santa Clara, CA, USA) with a 5 μm C18 XDB column (Agilent). The mobile phase consisted of 22.5% formic acid, 22.5% distilled water (DW), 45% acetonitrile, and 10% methanol. The 5 μL injection volume was allowed to flow at a rate of 1 mL/min at a temperature of 30°C. Decursin (standard) was procured from Sigma-Aldrich (St Louis, MO, USA) and analyzed at 530 nm.

### Human umbilical vein endothelial cell culture

Human umbilical vein endothelial cells (HUVECs) were purchased from ATCC (Manassas, VA, USA) and maintained in EBM2 medium (Lonza, Basel, Switzerland) under standard culture conditions. Upon reaching 85–90% confluence, the cells were incubated with AGE, or decursin for 48 h.

### Experimental animal protocols

Fifty 8-week-old male Sprague-Dawley rats weighing ~260 g were purchased from Orient Bio Inc., (Seongnam, Republic of Korea). All the rats were maintained under standard conditions with a 12 h L/D cycle and 55–60% relative humidity. After a week of acclimatization, the rats were divided into five groups of 10 rats each. This study consisted of five groups. Group 1, normal chow diet (NCD) group, received DW; Group 2, vehicle group, received HFD and DW; Group 3, AGE100, received HFD and 100 mg/kg of AGE; Group 4, AGE 200, received HFD and 200 mg/kg of AGE; and Group 5, AGE300, received HFD and 300 mg/kg of AGE. The AGE was dissolved in 0.9% saline and administered orally once daily for 8 weeks. The body weight of all experimental rats was assessed once a week during the intervention period. After 8 weeks, all the rats were euthanized for serum collection and tissue sampling. HFD (D12492, Research Diets Inc., New Brunswick, NJ) used in the study has protein (26.2 gm%), carbohydrate (26.3 gm%) and fat (34.9 gm%). Some of the key ingredients used in the HFD are casein, L-cysteine, corn starch, maltodextrin 10, sucrose, cellulose (BW200), soybean oil, lard, mineral mix, dicalcium phosphate, calcium carbonate, potassium citrate, vitamin mix, choline bitartrate and FD&C blue dye. To prepare the HFD, all dry ingredients were thoroughly mixed, and melted lard and soybean oil were added to the dry mixture. Next, the mixture was pelleted, dried and stored at room temperature.

### Quantification of nitric oxide levels in endothelial cells

Diamino-fluorescein-2-diacetate (DAF-2DA), a fluorescent NO probe (Molecular Probes, Eugene, OR, USA), was used to quantify the NO levels in human umbilical vein endothelial cells (HUVECs). Briefly, HUVECs were treated with DAF-2DA and incubated under normal culture conditions at 37°C for 30 min. Next, the fluorescence intensity (FI) emitted by DAF-2DA was recorded using EVOS M5000 fluorescence microscopy (Thermo Fisher Scientific, Waltham, MA, USA).

### Analysis of ROS production

ROS including O^2−^ levels were determined using dihydroethidium (DHE), an oxidation-sensitive fluorescent probe dye. The washed cells and aortic rings were treated with 20 μM DHE (Molecular Probes). Separately, 5 μM MitoSox (superoxide indicator; Molecular Probes) was treated to cells and aortic rings for analysis of O^2−^ levels. The cells and aortic rings were observed using an EVOS M5000 fluorescence microscopy (Thermo Fisher Scientific). The excitation/emission wavelengths for measuring the DHE and MitoSox fluorescence were 518/605 and 396/610 nm.

### Analysis of peroxynitrite production

Dihydrorhodamine (DHR) oxidation was used to determine the production of peroxynitrite by aortic rings. DHR oxidation was measured as previously described [39]. Embedded tissues were exposed to DPBS having 20 μM DHR (Invitrogen, Carlsbad, CA, USA) at 37°C for 30 min. The samples were observed using EVOS M5000 fluorescence microscopy (Thermo Fisher Scientific). The DHR oxidation product, rhodamine 123, was measured at a wavelength of 485/520 nm.

### Analysis of vascular function

After 8 weeks of the intervention, rats were anaesthetized using isoflurane (induction at 5%, then maintenance at 2.5% in 100 % oxygen) and euthanized by terminal exsanguination to minimize pain or distress. Subsequently, the thoracic aorta was quickly isolated and placed in ice-cold physiological salt solution (PSS) consisting of 119 mM NaCl, 4.7 mM KCl, 1.18 mM KH_2_PO_4_, 1.17 mM MgSO_4_∙7H_2_O, 24.9 mM NaHCO_3_, 1.6 mM CaCl_2_∙2H_2_O, 0.023 mM EDTA and 5.5 mM glucose. The isolated aorta was carefully separated from adherent connective tissue and fat using a stereomicroscope and then cut into 3–4 mm long ring segments. These segments, or aortic rings, were individually suspended in organ baths containing 5 mL of PSS. The solution was constantly bubbled with 95% O_2_ and 5% CO_2_, and maintained at a temperature of 37°C with a pH of 7.4. Each aortic ring was attached to a force transducer in the myograph to measure isometric tension. Rings were allowed to equilibrate for approximately 60 min under a resting tension of 1 g before starting the experimental protocols. The bath solution was changed every 10 min during the equilibration period. The segments were first contracted using 10^−6^ M phenylephrine (Phe). Various concentrations of acetylcholine (Ach) were treated to the bath for relaxation. The cumulative concentration-response curves from 10^−9^ M to 10^−6^ M ACh were plotted on the aortic rings. The isometric tension of the Ach-treated aortic rings was evaluated with a myograph using a PowerLab 8/35 data acquisition system (ADInstruments Pvt Ltd., Castle Hill, Australia).

### Analysis of lipid peroxidation

Lipid peroxidation produces reactive aldehydes such as malondialdehyde (MDA) and 4-hydroxynonenal (4-HNE). MDA and 4-HNE levels were quantified using a commercially available Bioxytech LPO-586 colorimetric assay kit (Oxis International Inc., Portland, OR, USA) to assess lipid peroxidation.

### Immunoblotting

Immunoblotting was performed as described previously [40]. Briefly, thoracic aorta tissue and HUVEC lysates were sonicated, separated on a polyacrylamide gel and transferred onto PVDF membranes. Blocked membranes were proved with primary antibodies against p-eNOS (#9571, Cell Signaling Technologies, USA), eNOS (#32027, Cell Signaling Technologies), nitrotyrosine (#sc32757, Santa Cruz Biotechnologies, USA), p-IRE1α (#ab124945, Abcam, UK), IRE1α (#3294, Cell Signaling Technologies), sXBP-1 (#ab220783, Abcam), GRP78 (#sc166490, Santa Cruz Biotechnologies), CHOP (#2895, Cell Signaling Technologies), Sirtuin 1 (#8469, Cell Signaling Technologies), p-AMPK (#2535, Cell Signaling Technologies), AMPK (#5832, Cell Signaling Technologies), and β-actin (#sc47778, Santa Cruz Biotechnologies). Blots were washed with TBS-T buffer, blocked with 5% Skim milk or BSA during 1 h and probed again with species-specific horseradish peroxidase conjugated secondary antibodies, anti-rabbit HRP 2nd antibody specifically for p-eNOS, eNOS, p-IRE1α, IRE1α, sXBP-1, CHOP, Sirtuin 1, p-AMPK, and AMPK and anti-mouse HRP 2nd antibody specifically for nitrotyrosine, GRP78 and β-actin. Protein signals were developed using ECL (Bio-Rad, Hercules, CA, USA), and their quantifications were performed by measuring the band intensity using the Image J analysis system (NIH, Bethesda, MD, USA).

### IRE1α sulfonation

Sulfonation was performed as was previously reported [33]. Total thoracic aorta tissues and HUVECs (~500 μg) were prepared using lysis buffer (Cell Signaling Technologies) containing protease and phosphatase inhibitors (Sigma-Aldrich). Immunoprecipitation was performed with anti-cysteine-sulfonate (#ab176487, Abcam), followed by an anti-IRE1α antibody (#3294, Cell Signaling) to detect IRE1α sulfonation. Protein A/G Sepharose beads (Sigma-Aldrich) were added and incubated for 1 h. Immunoprecipitates were washed 5 times with PBS-T buffer or PBS, separated using SDS-PAGE, and probed with specific antibodies.

### Real-time PCR analysis

Real-Time polymerase chain reaction assay (RT-PCR) was performed as described previously [21]. Briefly, homogenized thoracic aorta tissue and HUVECs were used for RNA extraction using the TRIzol solution (Invitrogen). Isolated RNA (2 μg) was used for cDNA synthesis using oligo dT primers and reverse transcriptase. The reaction mixture was prepared according to the manufacturer’s instructions (TaKaRa SYBR Premix Ex Taq Kit, TaKaRa Bio Inc., Tokyo, Japan). The reaction was performed on an ABI 7500 Real-Time PCR system (Applied Biosystems, Foster City, CA, USA). The amplified products were quantified using the comparative cycle threshold (Ct) method. Primers used in this study are listed in Table 1.

**Table 1:** Sequences for primers used in real-time RT-PCR.

**Genes**	**Forward (5′ to 3′)**	**Reverse (5′ to 3′)**
*hblos1*	TACAGGTCCAGCTGCCCAATT	TTCCAGTGCAGTGGCAATGGTG
*hhgsnat*	GGAGACGATCACCTTTACCAGC	TCACGATGGAGTTGATGGTGCC
*hcol6*	GCCTTCCTGAAGAATGTCACCG	TCCAGCAGGATGGTGATGTCAG
*hGAPDH*	GTCTCCTCTGACTTCAACAGCG	ACCACCCTGTTGCTGTAGCCAA

### Biochemical analyses

High-density lipoprotein (HDL, # XSYS0043), low-density lipoprotein-cholesterol (LDL-CHO, #XSYS0044), triglyceride (TG, #AM202K), total cholesterol (TC, #AM202K), alanine aminotransferase (ALT, #AM101-2), and aspartate aminotransferase (AST, #AM101-1) levels in the serum were analyzed using commercially available kits from Asan Pharm (Seoul, Republic of Korea).

### Histological staining

The tissues were fixed, embedded, and sectioned into 4 μm sections. Next, the sections were deparaffinized with xylene and rehydrated, then immunostained with anti-p-eNOS antibody and incubated with a TRITC-conjugated goat anti-mouse IgG (H+L) secondary antibody (#A16071, Thermo Fisher Scientific). Nuclei were stained with DAPI. The results were visualized using EVOS M5000 fluorescence microscope (Thermo Fisher Scientific). ImageJ analysis was used to analyze the mean fluorescence intensity. The images shown in this study were magnified at 40X.

### Statistical analysis

All statistical calculations and relevant analyses were performed using GraphPad Prism version 8.0 (GraphPad Software, San Diego, CA, USA). Data are shown as the mean ± SEM. One-way analysis of variance (ANOVA) with Tukey’s post-hoc test was used for multiple comparisons. *P* < 0.05 was considered statistically significant.

### Data availability statement

The data presented in this study are available upon request from the corresponding author.

## Supplementary Materials

Supplementary Figures
