# Supplementary material for: Angelica gigas extract inhibits acetylation of eNOS via IRE1α sulfonation/RIDD-SIRT1-mediated posttranslational modification in vascular dysfunction
Source: Aging (Albany NY). 2023 Dec 13;15(23):13608–27. doi: 10.18632/aging.205343 (PMC10756119; doi:10.18632/aging.205343)
Supplement: Supplementary Figures [file aging-15-205343-s001.pdf]

SUPPLEMENTARY FIGURES

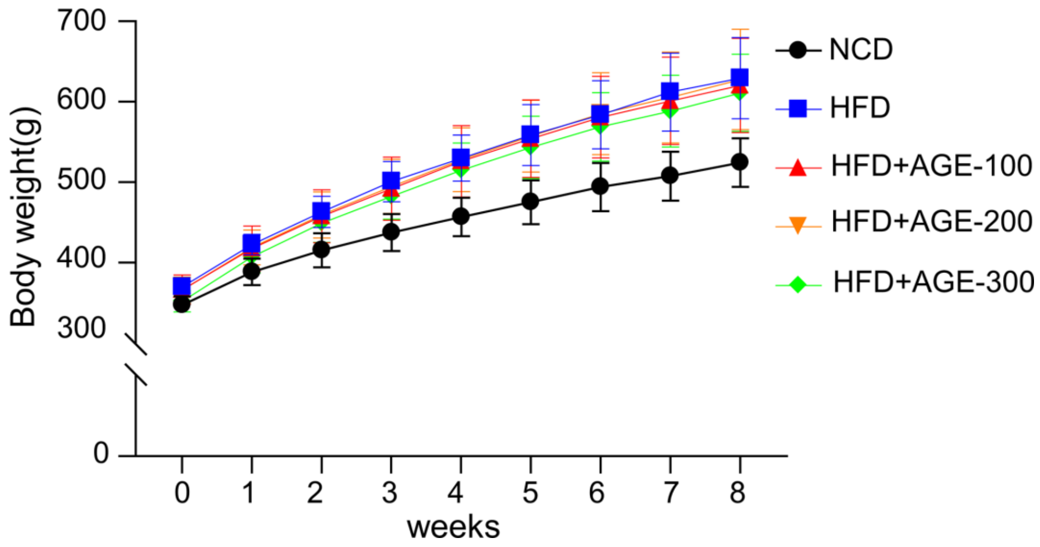

Supplementary Figure 1. Body weight and composition after 8 weeks of high-fat diet and AGE supplementation. Weekly body weight registration. Abbreviations: NCD: normal chow diet; HFD: high fat diet; AGE: *Angelica gigas NAKAI* extract.

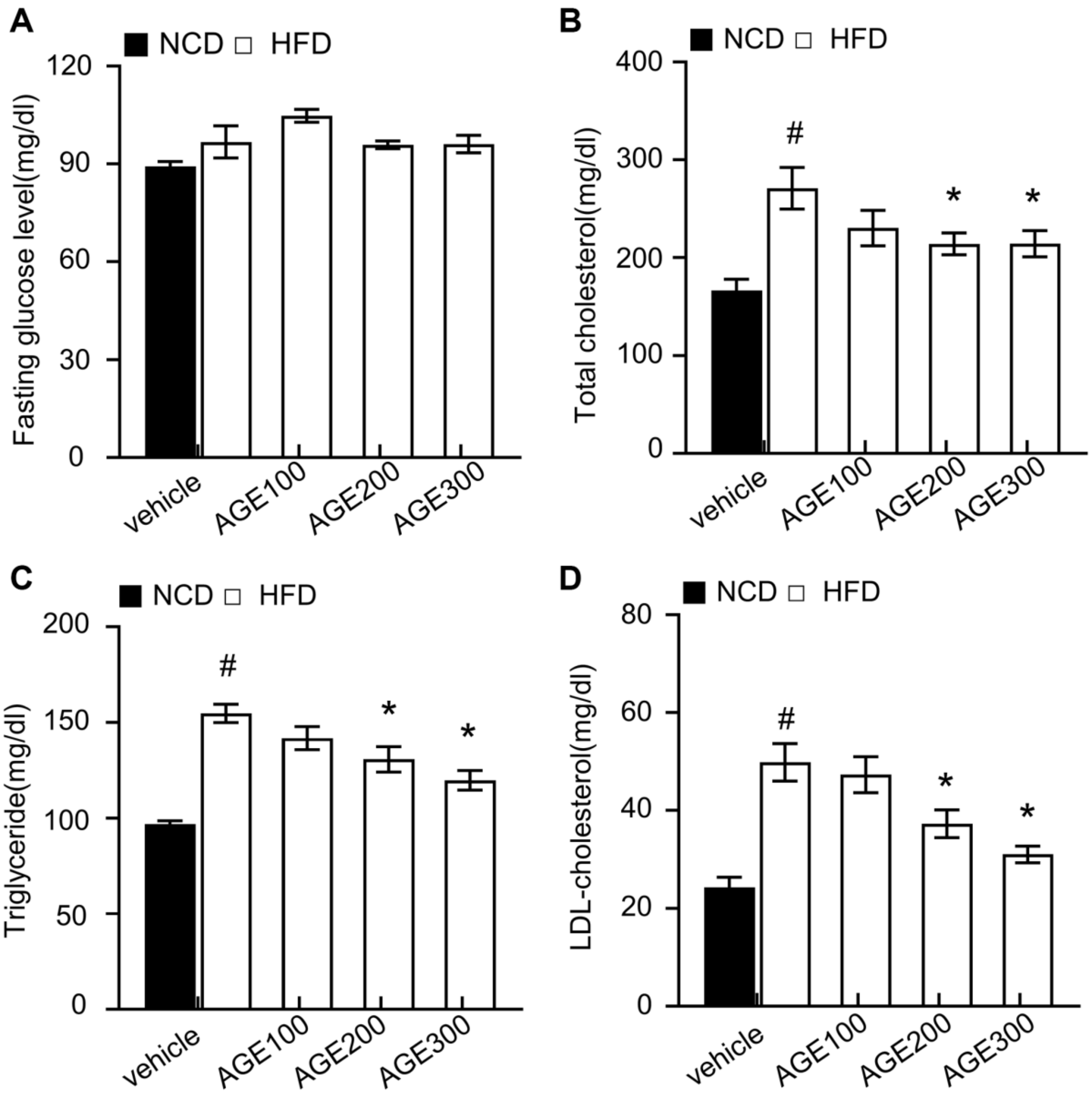

Supplementary Figure 2. AGE prevents high-fat diet-induced lipid dysmetabolism. Rats were fed normal chow diet or a high-fat diet with or without 100, 200 and 300 mg/kg AGE for 8 weeks, and serum was harvested. (**A**) Fasting glucose levels. (**B**) Levels of total cholesterol, (**C**) triglyceride and (**D**) low-density lipoprotein (LDL)-cholesterol were measured in the serum of rat in different experimental groups. Values are presented as mean ± SEM. (n = 8, $^{\#}p < 0.05$ versus the normal chow diet (NCD) group, $^{*}p < 0.05$ versus the HFD group). Abbreviations: NCD: normal chow diet; HFD: high fat diet; AGE: *Angelica gigas NAKAI* extract.

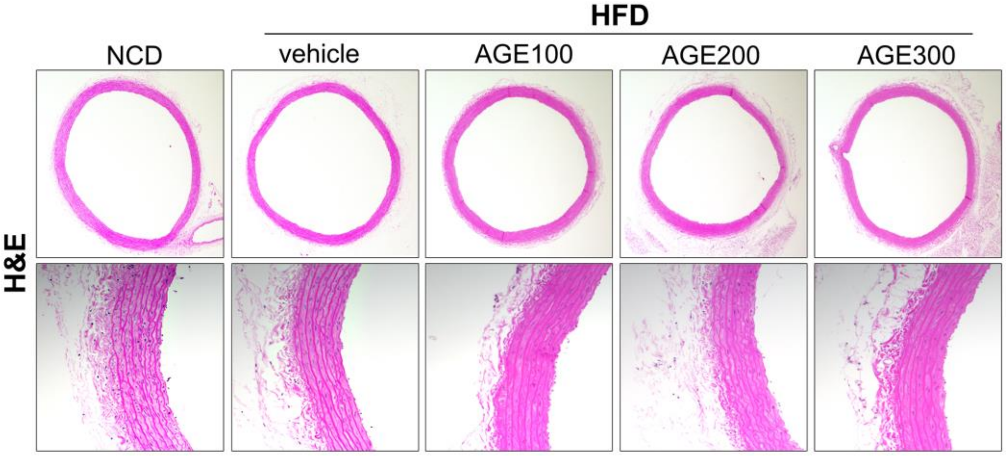

Supplementary Figure 3. Aorta rings tissues retrieved 8 weeks after initial AGE administration were subjected to hematoxyline-eosin (H&E) staining. Abbreviations: NCD: normal chow diet; HFD: high fat diet; AGE: *Angelica gigas NAKAI* extract.

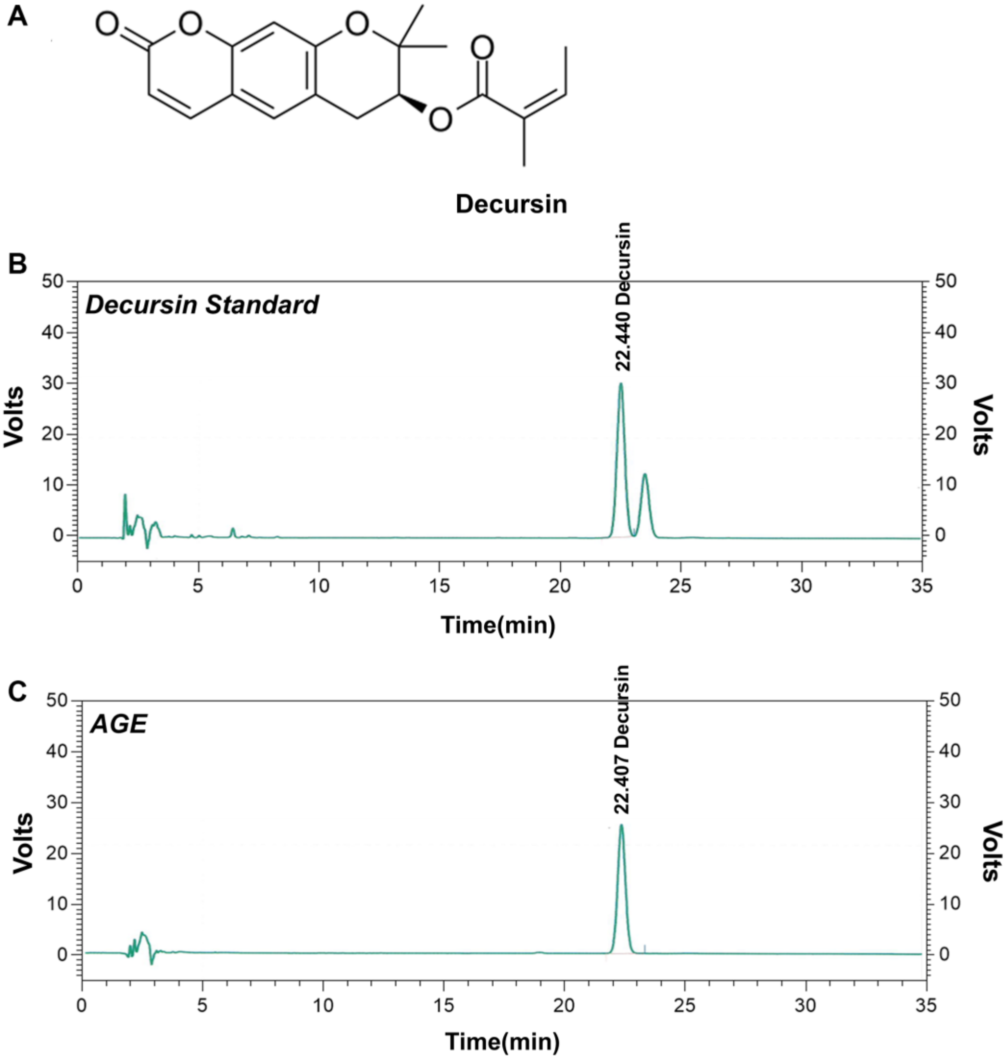

Supplementary Figure 4. HPLC analysis of AGE. (**A**) Chemical structure of decursin. (**B**) Chromatograms of decursin standard (**C**) and that of AGE analyzed for decursin.
